# Huaier Granule Combined with Tegafur Gimeracil Oteracil Potassium Promotes Stage IIb Gastric Cancer Prognosis and Induces Gastric Cancer Cell Apoptosis by Regulating Livin

**DOI:** 10.1155/2020/2403595

**Published:** 2020-07-08

**Authors:** Jing Qi, Fu-juan Xie, Sheng Liu, Cheng-yu Yao, Wei-hang Liu, Gao-qiang Cai, Guo-qing Liao

**Affiliations:** ^1^Department of Gastrointestinal Surgery, Xiangya Hospital of Central South University, Changsha, Hunan 410008, China; ^2^Department of Operating Room, Xiangya Hospital of Central South University, Changsha, Hunan 410008, China

## Abstract

Gastric cancer is one of the most common malignancies worldwide, with high morbidity and poor survival rate. Its prognosis remains unsatisfactory, with a 5-year survival rate of <30%. Studies have indicated that Huaier granules have good antitumor efficacy and safety in several solid malignant tumors. Recent studies have also found that Huaier polysaccharides can promote apoptosis in numerous tumor cells, although only few studies have focused on the effects of Huaier granules on gastric cancers and the mechanisms underlying their antitumor role. We retrospectively evaluated stage IIb gastric cancer patients at Xiangya Hospital, Central South University, through our outpatient system from January 2013 to December 2015. Fifty-four patients were in the Huaier+Tegafur Gimeracil Oteracil Potassium (TGOP) group and 72 in the TGOP group. Further, we conducted CCK8, colony formation, Annexin V-FITC/PI, Western blot, RT-PCR, and plasmid transfection assays to analyze the mechanism by which Huaier polysaccharides play an antitumor role. We confirmed that Huaier granules combined with Tegafur Gimeracil Oteracil Potassium could promote patient prognosis, with a better disease-free survival rate (51.32 ± 2.23 vs. 44.19 ± 2.26, *p* = 0.034) and overall survival rate (56.81 ± 1.32 vs. 51.32 ± 1.69, *p* = 0.020). Moreover, through cell proliferation assays, Western blot, RT-PCR, and detection of Livin expression at the mRNA and protein levels, we found that Huaier polysaccharides could promote gastric cancer cell apoptosis and inhibit gastric cancer cell proliferation in a time- and dose-dependent manner. Finally, we demonstrated that Huaier polysaccharides promote gastric cancer cell apoptosis through the regulation of Livin expression. Overexpression of Livin reversed the gastric cell apoptosis induced by Huaier polysaccharides. Huaier granules combined with Tegafur Gimeracil Oteracil Potassium ameliorated stage IIb gastric cancer prognosis and induced gastric cancer cell apoptosis by regulating Livin.

## 1. Introduction

Gastric cancer is one of the most common malignancies and is associated with high morbidity and poor survival rate worldwide, especially in eastern Asia [1, 2]. Despite much research focusing on the molecular mechanism and antitumor synthetic treatments (e.g., radical gastrectomy, chemotherapy, and radiotherapy) applied in gastric cancer, its prognosis remains unsatisfactory, with a 5-year survival rate lower than 30% [3]. Moreover, adjuvant postoperative chemotherapy or chemoradiotherapy for stages II, III, and IV is strongly recommended by the National Comprehensive Cancer Network (NCCN) and European Society for Medical Oncology (ESMO) guidelines [4, 5]. Even so, approximately 60% of the Western world and USA patients have got a 5 deaths a year recurrence rate [6]. Therefore, novel therapeutic agents with superior efficacy are urgently required for the clinical treatment of advanced gastric cancer.

*Trametes robiniophila Murr* (Huaier) is a type of sandy beige fungus that exists in China and has been extensively applied in many diseases as a traditional Chinese herbal medicine for over 1600 years [7]. Recent clinical studies indicated that Huaier granules have a good antitumor efficiency and safety in several solid malignant tumors, including hepatic carcinoma and breast cancer [8, 9]. Moreover, Huaier extract proteoglycans comprised 41.53% of polysaccharides, 12.93% of amino acids, and 8.72% of water analyzed using high-performance liquid chromatography [10]. In addition, a large number of studies have reported that Huaier extract, the Huaier polysaccharide, can enhance the immunity, antioxidation, and anti-inflammatory responses and downregulate Metadherin (MTDH) expression, which finally induces apoptosis in MCF-7 breast cancer cells [11].

Livin was first found in melanoma and belongs to the inhibitor of apoptosis protein (IAP) family. It inhibits cell proliferation by combining caspases 3, 7, and 9 with its BIR structural domain and RING domain to control the caspase cascading activation reaction [12]. Recent studies have demonstrated that Livin regulates cell progression, development, and apoptosis in many solid tumors through regulation of H2A.XY39ph and activation of epithelial-mesenchymal transition (EMT) [13].

Recent studies have found that Huaier polysaccharides can promote apoptosis of several tumor cells. We hypothesized that Huaier could promote the prognosis of gastric cancer patients by inhibiting cell apoptosis. In our study, we retrospectively evaluated stage IIb gastric cancer patients who received Tegafur Gimeracil Oteracil Potassium (TGOP) with/without Huaier granule as postoperative chemotherapy and aimed to determine the antitumor role of Huaier granules and the potential antitumor mechanism of Huaier polysaccharides in gastric cancer.

## 2. Materials and Methods

### 2.1. Patient Eligibility and Data Collection

We retrospectively evaluated stage IIb gastric patients through our outpatient system from January 2013 to December 2015 at Xiangya Hospital, Central South University. Additional clinical information and clinical follow-up dates were collected by our clinical system and the connection to patients. Eligibility criteria included (1) patients (age ≥ 18 and ≤75 years) with a confirmed first diagnosis of gastric cancer after R0 resection (D2 lymph node resection) and with their pathology and clinical data showing stage IIb patients according to the TNM stage, (2) patients with no other synchronous malignant neoplasia, and (3) patients simply orally administered Tegafur Gimeracil Oteracil Potassium Capsule with/without Huaier granule as postoperative chemotherapy, according to clinical guidelines [3].

Exclusion criteria included the following: (1) serious myelosuppression or liver and renal dysfunction, (2) combination with platinum drugs as postoperative chemotherapy, (3) patients who disregarded the doctors' advice or have irregular medication of taking chemotherapy, (4) irritability to any of the above drugs, (5) women who were pregnant or breastfeeding, and (6) patients who had received preoperative radiotherapy, chemotherapy, or other antitumor medications before our study.

Patients received oral Tegafur Gimeracil Oteracil Potassium Capsule 60 mg twice daily, from day 1 to day 14, three weeks of a cycle with a duration of one year, with/without Huaier granule 20 mg three times a day for a duration of one year.

All patients provided written consent to participate in the project. The research received support from the Medical Ethical Committee of Xiangya Hospital, Central South University (No. 201912535).

### 2.2. Chemicals and Reagents

Huaier granules were obtained from Qidong Gaitianli Pharmaceutical, Jiangsu, China, and Tegafur Gimeracil Oteracil Potassium Capsule was obtained from Qilu Pharmaceutical Co. Ltd. Huaier purification (named Huaier polysaccharide (HP)) was donated from Qidong Gaitianli Pharmaceutical, Jiangsu, China; DMEM was purchased from HyClone, Logan, UT, USA. FBS was purchased from Gibco, Life Technologies, USA; penicillin was purchased from Invitrogen, Shanghai, China; artemisinin, dimethyl sulfoxide (DMSO), and cell counting kit 8 (CCK8) were bought from Sigma, St. Louis, MO, USA; FITC and FITC anti-mouse/rat CD29 were bought from eBioscience, San Diego, CA, USA; Annexin V-FITC/PI Kit was bought from Sangon Biotech, Shanghai, China; caspases 3, 8, and 9 and Livin antibody were purchased from Abcam, Cambridge, UK.

### 2.3. HP Isolation and Purification

HP was isolated and purified as previously described [13]. Briefly, the first incipient HP was obtained by dehydration and distillation of the fruiting bodies; then, a diethylaminoethyl (DEAE) cellulose-52 column chromatography was conducted, washed with distilled water, and eluted with 0.1-0.3 mol/L sodium chloride. Finally, the purity of HP was evaluated by using a phenol-sulfuric acid method with glucose as the standard [14].

### 2.4. Cell Culture and Transfection

Gastric cancer cell lines SGC-7901 and BGC-823 were obtained from the Chinese Academy of Sciences (Shanghai, China) and maintained in Dulbecco's modified Eagle's medium (DMEM) containing 10% fetal bovine serum (FBS), 100 mg/mL streptomycin, and 100 U/mL penicillin in humidified air at 37°C with 5% CO_2_. SGC-7901 cell lines were seeded at a density of 1 × 10^5^ cells per well in 6-well plates and transfected with the *livin* gene using Lipofectamine 2000 (Life Technologies Inc.), according to the manufacturer's instructions. The primers used to amplify the *livin* gene were as follows: forward 5′-GGCTGCGTCTTCCGGTTCTT-3′ and reverse 5′-GGTGAGGTGCTTCTTCTGCTATGG-3′, for the internal control gene GADPH, forward 5′-CCAGGTGGTCTCCTCTGA-3′ and reverse 5′-GCTGTAGCCAAATCGTTGT-3′.

### 2.5. Cell Counting Kit 8 (CCK8) Assay

Cell proliferation was monitored using a CCK8 kit, following the manufacturer's instructions. Briefly, cells with different concentrations of Huaier polysaccharide (0, 50, and 100*μ*g/mL) were cultivated in five 96-well plates with six replicate wells, and gastric cancer cells (SGC-7901, BGC-823) were transfected with the Livin mimic combined with Huaier polysaccharide in rescue experiments.

### 2.6. Colony Formation Assay

For the colony formation assay, 400-1000 transfected cells were plated in a 6-well plate and kept in medium with 10% FBS for 2 weeks with the medium replacements every 3 days. Colonies were then confirmed with methanol and dyed using 0.1% crystal violet (Sigma-Aldrich, St Louis, MO) for 30 min. Visible stained colonies were counted in the colony formation analysis. In different treatment groups, the wells were independently measured in triplicates.

### 2.7. Annexin V-FITC/PI Assay

The apoptotic rates were analyzed using the FACS methodology recommended in the Annexin V-FITC/PI Kit. Briefly, SGC-7901 cells were plated into 6 wells (1 × 10^6^ cells/mL). After the treatment with different concentrations of Huaier polysaccharide or transfection with Livin mimic and Huaier polysaccharide and different durations, the cells were harvested and washed with the binding buffer, suspended in 200 *μ*L of binding buffer at a cell density of 5 × 10^5^ cells/mL, incubated with Annexin V-FITC (10 *μ*g/mL, 5 *μ*L), and incubated at 25°C for 15 min in the dark. At the end of incubation, SGC-7901 cells were washed with the binding buffer (500 *μ*L) and centrifuged at 2000 rpm for 5 min. Cells were suspended in 190 *μ*L of binding buffer with 10 *μ*L of PI (20 *μ*g/mL) and lucifuge at 25°C for 15 min. The samples were acquired in BD FACSCalibur within 1 h, and the collected data were analyzed using FlowJo 7.6. The apoptosis rate was expressed as the percentage of Annexin V-positive cells.

### 2.8. Western Blotting

Western blot analyses were performed using standard methods. Briefly, cell protein lysates were separated using a 12% SDS-PAGE, transferred to a PVDF transfer membrane (Millipore), blocked with 5% fat-free milk for 4 h at 20°C, cultivated with particular antibodies overnight at 4°C, washed with TBST five times, and incubated with the secondary antibody for 2 h. The proteins were visualized using a detection system of enhanced chemiluminescence (ECL) and exposed to X-ray film. *β*-Actin antibody was used as a control.

### 2.9. Quantitative Real-Time Polymerase Chain Reaction (qRT-PCR)

Total RNA was extracted from cells or tissues with a TRIzol reagent according to the manufacturer's instructions and quantified using a NanoDrop 1000 system (NanoDrop Technologies, Rockland, DE, USA). Total RNA integrity was evaluated using standard denaturing agarose gel electrophoresis. Reverse transcription for mRNAs was performed using PrimeScript™ RT Master Mix. The cDNA templates were amplified using RT-PCR using the SYBR Green PCR Kit (TaKaRa). The qRT-PCR reactions were run in triplicates using the ABI7500 System (Applied Biosystems, USA) and the accompanying analytical software. *β*-Actin was used as an internal control. The relative expression levels of lncRNA and mRNA were calculated using the 2-*ΔΔ*Ct method. The PCR primer sequences were as follows: caspase 8: forward, 5′-TCGACGATTACGAACGATCA-3′ and reverse, 5′-CAGTCTTTGCCCTTGTGGTC-3′; caspase 3: forward, 5′-AGTTGGACCCACCTTGTGAG-3′ and reverse, 5′-AGTCTGCAGCTCCTCCACAT-3′; Bcl-2: forward, 5′-CCTGTGGATGACTGAGTACCTGA-3′ and reverse, 5′-CAGAGACAGCCAGGAGAAATCA-3′; Bax: forward, 5′-GTTTCATCCAGGATCGAGCAG-3′ and reverse, 5′-CCATCTTCTTCCAGATGGTGAGT-3′; and *β*-actin: forward, 5′-AGCCATGTACGTAGCCATCC-3′ and reverse, 5′-CTCTCAGCTGTGGTGGTGAA-3′.

### 2.10. Statistical Analyses

The counting data are shown as percentages, and measurement data are shown as the mean or median with standard deviation (SD). Patient clinical characteristics between each group were compared using the chi-squared test or Fisher's exact test. Disease-free survival (DFS) and overall survival (OS) were obtained by using the Kaplan-Meier method, and differences between Kaplan-Meier curves were investigated using the log-rank test. Student's *t*-test or one-way ANOVA was used for comparison between different groups. Statistical analysis was performed using SPSS version V22.0 (SPSS Inc., USA). The value of *p* was considered to be statistically significant below the 5% level.

## 3. Results and Discussion

### 3.1. Huaier Granule Combined with Tegafur Gimeracil Oteracil Potassium Promotes Stage IIb Gastric Cancer Prognosis

**Figure 1:**
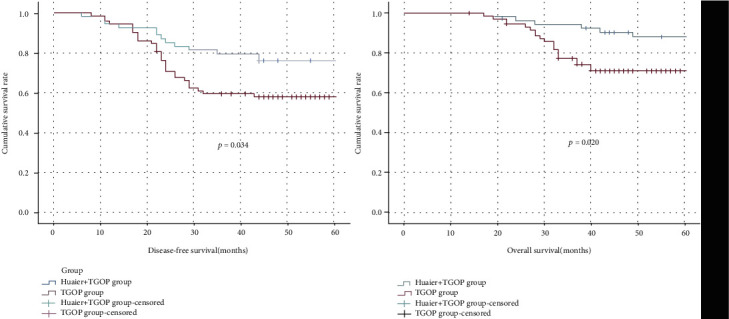
Disease-free survival (DFS) and overall survival (OS) for stage IIb gastric cancer patients stratified by different groups.

To investigate the antitumor function of Huaier granules, we retrospectively evaluated patients from January 2013 to December 2016 at Xiangya Hospital, who were diagnosed with stage IIb gastric cancer. The main postoperative chemotherapy regimens were simply taking Tegafur Gimeracil Oteracil Potassium Capsule with/without Huaier granule, orally. The clinical features and demography of these patients are shown in Table 1. Fifty-four patients were in the Huaier+Tegafur Gimeracil Oteracil Potassium group, and 72 patients were in the Tegafur Gimeracil Oteracil Potassium group. There was no statistical difference between age, sex, surgical methods, tumor size, tumor location, T stage, N stage, and perineuronal and vascular invasion between each group (*p* > 0.05). Moreover, through Kaplan-Meier curves (Figure 1), we investigated the disease-free survival (DFS) and overall survival (OS) and found that patients who orally took Tegafur Gimeracil Oteracil Potassium Capsule combined with Huaier granule got a better DFS (51.32 ± 2.23 vs. 44.19 ± 2.26, *p* = 0.034) and OS (56.81 ± 1.32 vs. 51.32 ± 1.69, *p* = 0.020) compared with simply oral Tegafur Gimeracil Oteracil Potassium Capsule, suggesting that Huaier granule combined with Tegafur Gimeracil Oteracil Potassium can help promote a stage IIb gastric cancer prognosis, which indicated that Huaier might play an antitumor role in gastric cancer therapy.

### 3.2. Huaier Polysaccharides Promote Gastric Cancer Cell Apoptosis with Dose and Duration Dependence

**Figure 2:**
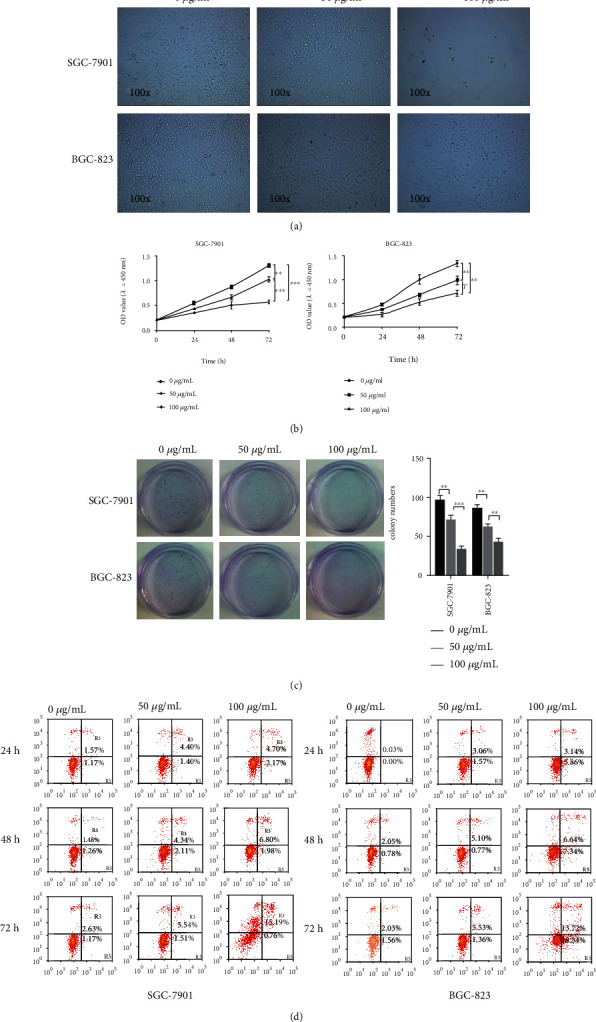
Huaier polysaccharides promote gastric cancer cell apoptosis with dose and duration dependence. (a) Cellular morphological evaluation of SGC-7901 and BGC-823 cells after treatment with 0, 50, and 100 *μ*g/mL of Huaier polysaccharide for 72 h, observed by using an inverted phase contrast microscope at a 100x magnification. (b) CCK assays showed the OD value (*λ* = 450 nm) after treatment with 0, 50, and 100 *μ*g/mL Huaier polysaccharide for 24, 48, and 72 h, respectively. (c) Colony formation assay analysis of SGC-7901 and BGC-823 cell proliferation after treatment with 0, 50, and 100 g/mL Huaier polysaccharide, respectively. (d) Annexin-FITC/PI assay and flow cytometry to analyze the SGC-7901 and BGC-823 cell apoptosis after treatment with 0, 50, and 100 *μ*g/mL Huaier polysaccharide for 24, 48, and 72 h, respectively.

To further investigate the antitumor mechanism of Huaier granules in gastric cancer, Huaier extract and purificatory production (named Huaier polysaccharide) were used to perform the following experiments. Inverted phase contrast microscopy was used to examine the effects of different doses of Huaier polysaccharide after treatment for 72 h on the cellular morphology in SGC-7901 and BGC-823 cells. The results indicated that, compared with the control group, cells treated with Huaier polysaccharide showed an obvious decrease and a significant detachment of cells from one another, forming a small cluster of cells with a dosage dependence (Figure 2(a)). A CCK8 assay was further conducted to analyze cell proliferation. The results showed that Huaier polysaccharide exhibited potent cytotoxic effects in the SGC-7901 and BGC-823 cell lines, in a dose- and time-dependent manner. Moreover, the results indicated that the maximum effect was observed at a dose of 100 *μ*g/mL of Huaier polysaccharide and for a processing time of 72 h, with inhibition ratios of 56.46 ± 3.07% and 46.60 ± 3.80% in SGC-7901 and BGC-823 cells, respectively (Figure 2(b)). Furthermore, the results of the colony formation assay showed that Huaier polysaccharide exhibited SGC-7901 and BGC-823 cell proliferation in a dose-dependent manner (Figure 2(c)). In addition, Annexin-FITC/PI assay and flow cytometry were performed to analyze cell apoptosis with different dose and time approaches, while applying Huaier polysaccharide. Results showed that, compared with the control group, the group treated with Huaier polysaccharide promoted apoptosis in gastric cancer cell lines in a dose- and time-dependent manner, and in SGC-7901 and BGC-823 cells applied at a dose of 100 *μ*g/mL Huaier polysaccharide, and for a processing time of 72 h, the greatest effect was observed, with an apoptosis rate of 15.95% and 32.06%, respectively (Figure 2(d)).

### 3.3. Huaier Polysaccharides Promote GC Cell Apoptosis via the Extrinsic Pathway (Death Receptor Pathway) and Intrinsic Pathways (the Mitochondrial Pathway)

**Figure 3:**
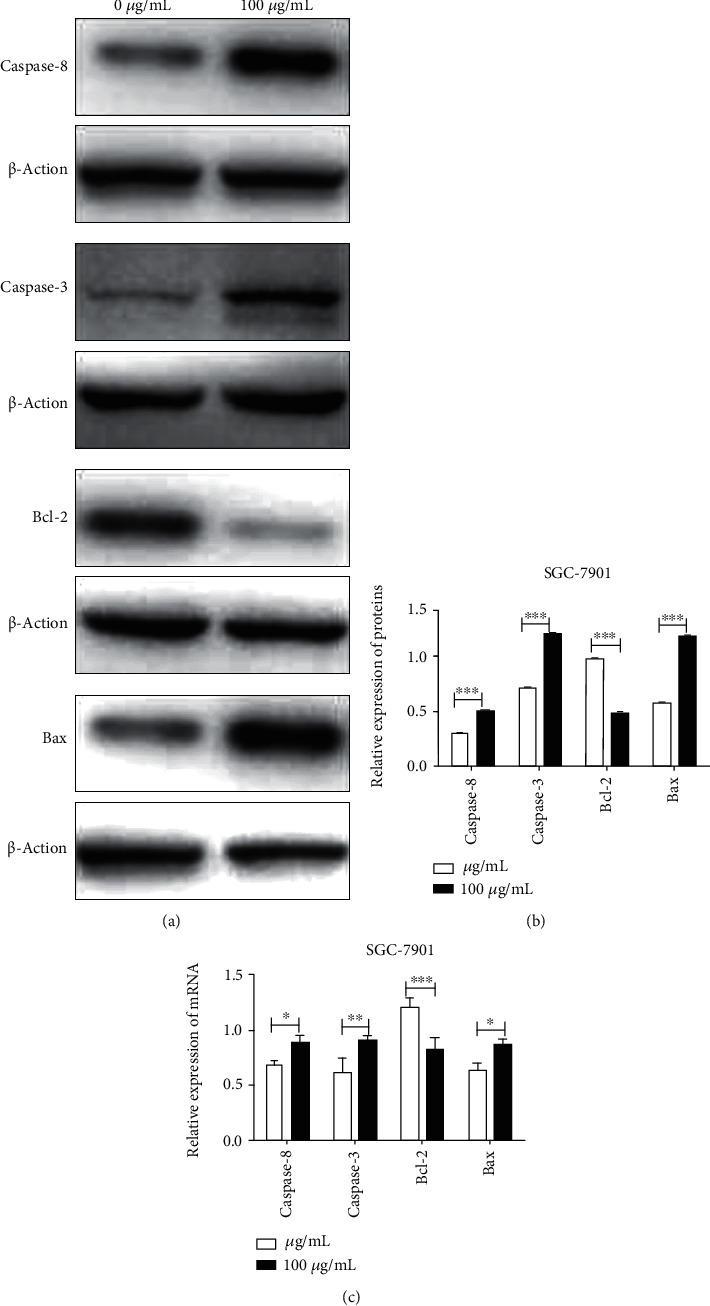
Huaier polysaccharides promote SGC-7901 cell apoptosis by activating the extrinsic pathway and intrinsic pathways. (a, b) Relative caspase 8, Bcl-2, Bax, and caspase 3 expression in SGC-7901 treated with 100 *μ*g/mL Huaier polysaccharide and processed after 72 h at the protein level. (c) Relative expression of caspase 8, Bcl-2, Bax, and caspase 3 in SGC-7901 treated with 100 *μ*g/mL Huaier polysaccharide and processed after 72 h at the mRNA level.

Further experiments were performed to explore the apoptotic pathways in cells treated with Huaier polysaccharide. SGC-7901 cells were treated with 100 *μ*g/mL Huaier polysaccharide and processed after 72 h. As shown in Figure 3, we tested caspase 8 expression both at the mRNA and protein levels, which could reflect whether it had activated the extrinsic apoptotic pathways. We also tested Bax and Bcl-2 expression at both the mRNA and protein levels, which could reflect whether it had activated the intrinsic apoptotic pathways. Finally, we tested the expression of cleaved caspase 3, which was the downstream target gene and promoted cell apoptosis. Results showed that the antiapoptotic protein Bcl-2 was frequently downregulated, and both caspase 8 and proapoptosis protein Bax were upregulated, together with their apoptotic effective protein cleaved caspase 3 at the mRNA and protein levels, while SGC-7901 cells were treated with Huaier polysaccharide. These findings indicated that both endogenous and exogenous apoptotic pathways were all activated to promote cell apoptosis while treated with Huaier polysaccharide.

### 3.4. Huaier Polysaccharides Promote SGC-7901 Cell Apoptosis by Regulating Livin

**Figure 4:**
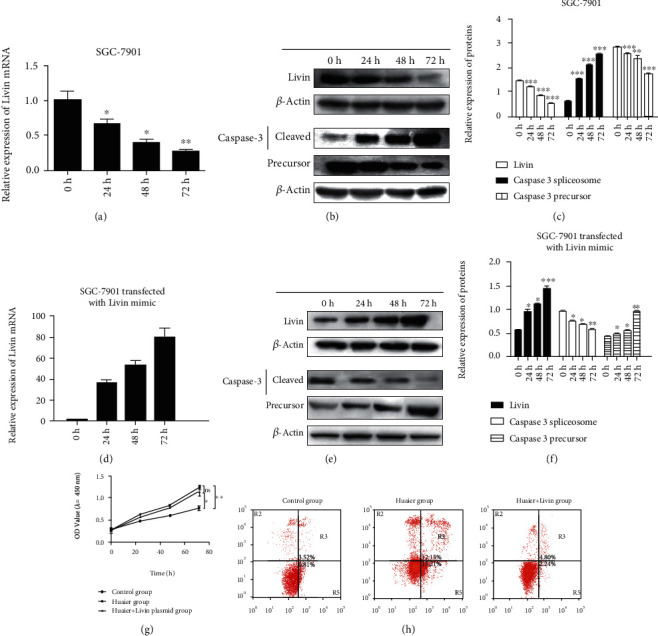
Huaier polysaccharides promote SGC-7901 cell apoptosis by regulating the expression of Livin. (a) Relative expression of Livin expression in SGC-7901 treated with 100 *μ*g/mL Huaier polysaccharide and processed after 72 h at the mRNA level. (b, c) Relative expression of Livin and caspase 3 expression in SGC-7901 treated with 100 *μ*g/mL Huaier polysaccharide and processed after 72 h at the protein level. (d) Relative expression of Livin in SGC-7901 cells transfected with the Livin mimic and treated with 100 *μ*g/mL Huaier polysaccharide, after 72 h at the mRNA level. (e, f) Relative expression of Livin expression in SGC-7901 cells transfected with the Livin mimic and treated with 100 *μ*g/mL Huaier polysaccharide, after 72 h at the protein level. (g) CCK8 assay analysis of the OD value (*λ* = 450 nm) in each group. (h) Annexin V-FITC/PI assay and flow cytometry to analyze the SGC-7901 cell apoptosis in each group and processing after 72 h.

To confirm the target gene in SGC-7901 cells treated with Huaier polysaccharide, we detected the Livin expression in SGC-7901 treated with 100 *μ*g/mL Huaier polysaccharide and processed after 72 h. Results showed that Livin expression was downregulated at the mRNA and protein levels while treated with Huaier polysaccharide, together with the downregulation of precursor of caspase 3 and upregulation of cleaved caspase 3 at the protein level (Figures 4(a)–4(c)). SGC-7901 cells were transfected with the Livin plasmid, treated with 100 *μ*g/mL Huaier polysaccharide, and processed after 72 h. Results showed that Livin expression was rescued while transfected with the Livin plasmid at the mRNA and protein levels, together with the precursor of caspase 3 at the protein level, and cleaved caspase 3 was downregulated while transfected with the Livin plasmid (Figures 4(d)–4(f)). In addition, we conducted other CCK8 assays, Annexin -FITC/PI assay, and flow cytometry to investigate the cell proliferation and apoptosis effects when cells were transfected with Livin and treated with Huaier polysaccharide, as shown in Figures 4(g) and 4(h). Overexpression of Livin could help rescue SGC-7901 cell apoptosis induced by Huaier polysaccharide (apoptosis ratio from 27.34 to 7.04%), indicating that Huaier polysaccharide promoted SGC-7901 cell apoptosis and inhibited SGC-7901 cell proliferation by targeted Livin expression.

## 4. Discussion

Despite the declining incidence of gastric cancer, it remains the fifth most common cancer diagnosis and the third most common cause of cancer-related mortality, having been responsible for over 1,000,000 new cases and estimated 783,000 deaths in 2018 [15], which reflects an advanced disease at presentation and aggressive biology. Numerous studies have proved that postoperative strategies, including chemotherapy, can clearly improve the outcomes. However, most drugs applied for chemotherapy and anticancers can cause irreversible damage, including liver, kidney, and immune system damage, indicating that more beneficial strategies should be explored to improve the outcome and alleviate the cytotoxicity to normal organs and the immune system. Compared with cytotoxicity to cancer cells, inducing cancer cell apoptosis might be a better strategy for anticancer treatment. Huaier, a traditional Chinese medicine, has been widely applied due to its anti-inflammatory and anticancer properties. Our research has shown that Huaier combined with Tegafur Gimeracil Oteracil Potassium Capsule as a postoperative anticancer therapy could get a better benefit in gastric cancer patients. Moreover, Huaier extract and Huaier polysaccharide could decrease gastric cancer cell proliferation and induce gastric cancer cell apoptosis by regulating the expression of Livin, which might help us find new target genes in anticancer therapy.

Huaier has been extensively used as an antitumor drug in the past 1600 years [10]. A multicenter, randomized clinical trial by Chen et al. has proved a significant prolongation of recurrence-free survival and reduced extrahepatic recurrence rates in hepatocellular carcinoma (HCC) patients taking Huaier granules, providing evidence that Huaier granules could be used as a postoperative adjuvant therapy for HCC [8]. Another study by Fang et al. also found that patients orally administrated Huaier granules had a longer DFS, indicating that Huaier could prolong the DFS of malignant tumor patients and play an important role in anticancer therapy [16]. Our research also found that patients who orally took Huaier granule combined with Tegafur Gimeracil Oteracil Potassium Capsule could significantly prolong the DFS and OS in gastric cancer stage II, comparing with patients who only orally took Tegafur Gimeracil Oteracil Potassium Capsule. Although it was a retrospective study from a single center, it could still indicate that Huaier granule is efficient in the treatment of gastric cancer.

To investigate the mechanisms by which Huaier granules play an antitumor role in gastric cancer, Huaier polysaccharide, the purification result of Huaier granules and the most important ingredient in Huaier granules, was obtained in our research. In addition, Li et al. found that Huaier polysaccharides could inhibit HCC growth and metastasis by suppressing angiogenesis *in vitro* and *in vivo* [17]. Luo et al. also indicated that Huaier polysaccharide induced MCF-7 breast cancer cell apoptosis by downregulating the expression of MTDH protein [11]. Fang et al. also demonstrated that Huaier polysaccharides inhibited renal cancer cell proliferation, metastasis, and invasion by inducing apoptosis and inhibiting multiple signaling pathways, including P13K/Akt/VEGFRA signaling, and suppressing EMT [16]. However, the mechanisms by which Huaier polysaccharides play an anticancer role in gastric cancer remain to be explored. In our study, we found that Huaier polysaccharide inhibited the SGC-7901 and BGC-823 gastric cancer cell proliferation in a time- and dose-dependent manner by using a CCK8 assay. The colony formation assay also helped us identify the cytotoxicity and inhibitory effects of Huaier polysaccharides on SGC-7901 gastric cancer cells in a time- and dose-dependent manner. These effects rely on the stimulation of cell apoptosis.

Our research found that Huaier polysaccharides could induce SGC-7901 gastric cancer cell apoptosis, leading to the inhibition of SGC-7901 gastric cancer cell proliferation. Moreover, the Annexin V-FITC/PI assay showed that Huaier polysaccharide induced SGC-7901 gastric cancer cell apoptosis in a time- and dose-dependent manner, which resulted in the inhibition of SGC-7901 gastric cancer cell proliferation. Cells undergo apoptosis through two major pathways, the extrinsic pathway (death receptor pathway) or the intrinsic pathway (the mitochondrial pathway). Finally, cells are packaged into apoptotic bodies and recognized by neighboring cells or macrophages and cleared by phagocytosis [17]. In our research, we investigated the critical molecular marker caspase 8 expression in the extrinsic pathway and the expression of critical molecular markers Bcl-2 and Bax in the intrinsic pathway. As expected, both the extrinsic and the intrinsic pathways were activated to promote cell apoptosis.

Livin belongs to the IAP family with its BIR structural domain and a RING domain to control the caspase cascading activation reaction and plays a crucial role in cell apoptosis, proliferation, and cell cycle control [18]. Previous studies have confirmed that an overexpression of Livin in tumor tissues is a risk factor and a poor prognostic factor in malignant tumors. Livin was first analyzed by Vucic et al., who initially found that Livin was highly expressed in most melanoma cell lines and that an upregulation of Livin indicated resistance to apoptotic stimuli, thereby potentially contributing to the pathogenesis of this malignancy [18]. Zareifar et al. showed that a Livin upregulation in bone marrow blocks was associated with a poor prognosis in nonacute promyelocytic leukemia [19]. Similar results have been reported for most malignant tumors, including lung and colon cancers [20, 21]. Silencing of Livin is associated with activation and strongly increases the apoptotic rate in response to different proapoptotic stimuli, including doxorubicin, UV irradiation, or TNF-*α* [22]. These studies indicated that Livin silencing might be a potential therapeutic strategy in cancers. Our study showed that Huaier polysaccharide induced the apoptosis of gastric cancer cell SGC-7901. We further conducted Western blotting and qRT-PCR to analyze the expression of Livin and found that it was downregulated when cells were processed with Huaier polysaccharide, in a time- and dose-dependent manner at the mRNA and protein levels, and the downstream activation of target gene caspase 3 was also associated with the expression of Livin. Moreover, we analyzed the outcome while cells were processed combined with Huaier polysaccharide and Livin overexpression plasmid transfection, and CCK8 assays found that the exogenous upregulation of Livin reversed the inhibition of SGC-7901 cell proliferation induced by Huaier polysaccharide. In addition, from the Annexin V-FITC/PI assay, we found that an upregulation of SGC-7901 cell apoptosis was inhibited by Livin overexpression, indicating that Huaier polysaccharide regulated gastric cancer SGC-7901 cell proliferation and apoptosis by targeting Livin.

Our research has several limitations. It should be stressed that it was a retrospective study, so some confounding variables (such as BMI, smoking history, and dietary behavior) cannot be compared completely between each group, and further randomized controlled, multiple-center, and prospective studies should be performed. Besides, our research found that Huaier polysaccharide activated the extrinsic (death receptor pathway) and intrinsic (the mitochondrial pathway) pathways to promote SGC-7901 cell apoptosis and regulated Livin expression to inhibit cell proliferation and promote apoptosis; however, through CCK8 and Annexin-V FITC/PI assays and flow cytometry, we found that the overexpression of Livin could not completely reverse the effects when SGC-7901 gastric cancer cells were treated with Huaier polysaccharide, and recent studies showed that Livin regulates cell apoptosis mainly by activating the intrinsic pathway, but there might be other mechanisms via which Huaier polysaccharide promotes gastric cancer cell apoptosis. In addition, we found that Huaier granule combined with Tegafur Gimeracil Oteracil Potassium promotes the stage IIb gastric cancer prognosis, and there might be other mechanisms to explain the results; for example, Huaier polysaccharide helps to enhance the 5-Fu cytotoxicity to gastric cancer cells. Finally, mouse xenograft models should be used in future studies, and tumor growth should be observed i*n vivo* to further confirm our results.

## 5. Conclusions

Huaier granules combined with Tegafur Gimeracil Oteracil Potassium could promote stage IIb gastric cancer prognosis. Huaier polysaccharides inhibited the proliferation of gastric cancer SGC-7901 cells and induced SGC-7901 cell apoptosis by regulating Livin.

## Figures and Tables

**Table 1:** Gastric cancer patients' characteristics in different groups (*n* = 126).

Characteristics	Groups		*p* value
	Huaier+TGOP group (*n* = 54)	TGOP group (*n* = 72)	
Age (years)			0.454
<60	37	44	
≥60	17	28	
Gender			0.716
Male	32	45	
Female	22	27	
Methods of surgery			0.527
Open surgery	48	67	
Laparoscopic surgery	6	5	
Tumor size (cm)			0.159
<5	43	48	
≥5	11	24	
Location			0.139
Cardia	3	3	
Fundus	12	17	
Body	23	18	
Antrum	16	34	
T stage			0.811
T2-T3	8	12	
T4	46	60	
N stage			0.811
N0-N1	46	60	
N2	8	12	
Perineuronal invasion			0.203
Yes	49	59	
No	5	13	
Vascular invasion			0.181
Yes	47	57	
No	7	15	

TGOP: Tegafur Gimeracil Oteracil Potassium.

## Data Availability

The raw data underlying this paper are available upon request to the corresponding author due to ethical restrictions.

## References

[1] Karimi P., Islami F., Anandasabapathy S., Freedman N. D., Kamangar F. (2014). Gastric cancer: descriptive epidemiology, risk factors, screening, and prevention. *Cancer Epidemiology, Biomarkers & Prevention*.

[2] Zhang K., Liu W., Qu Z. (2019). *In vitro* and *in vivo* human gastric cancer inhibition by Trifolirhizin is facilitated via autophagy, mitochondrial mediated programmed cell death, G2/M phase cell cycle arrest and inhibition of m-TOR/PI3K/AKT signalling pathway. *Journal of BUON*.

[3] Yamamoto H., Watanabe Y., Maehata T. (2014). An updated review of gastric cancer in the next-generation sequencing era: insights from bench to bedside and vice versa. *World Journal of Gastroenterology*.

[4] Ajani J. A., D'Amico T. A., Almhanna K. (2016). Gastric cancer, version 3.2016, NCCN clinical practice guidelines in oncology. *Journal of the National Comprehensive Cancer Network*.

[5] Smyth E. C., Verheij M., Allum W. (2016). Gastric cancer: ESMO Clinical Practice Guidelines for diagnosis, treatment and follow-up. *Annals of Oncology*.

[6] Miller L. H., Kastin A. J., Sandman C. A. (1977). Psychobiological actions of MSH in man. *Frontiers of Hormone Research*.

[7] Zhang N., Kong X., Yan S., Yuan C., Yang Q. (2010). Huaier aqueous extract inhibits proliferation of breast cancer cells by inducing apoptosis. *Cancer Science*.

[8] Chen Q., Shu C., Laurence A. D. (2018). Effect of Huaier granule on recurrence after curative resection of HCC: a multicentre, randomised clinical trial. *Gut*.

[9] Clark R. G., Mason P. C., Fennessy P. F. (1978). Nodular lesions in the absence of Oesophagostomum columbianum. *New Zealand Veterinary Journal*.

[10] Guo Y., Chen P. (1993). Isolation and analysis of the polysaccharide of Huaier mycelium. *Chinese Journal of Biochemical Pharmaceutics*.

[11] Luo Z., Hu X., Xiong H. (2016). A polysaccharide from Huaier induced apoptosis in MCF-7 breast cancer cells via down-regulation of MTDH protein. *Carbohydrate Polymers*.

[12] Li W., Li X., Wang G., Fu B. (2011). Gene Expression of Livin and Survivin in Adult Patients With Acute Lymphoblastic Leukemia and Its Clinical Significance. *Zhongguo Shi Yan Xue Ye Xue Za Zhi*.

[13] Li F., Yin X., Luo X. (2013). Livin promotes progression of breast cancer through induction of epithelial- mesenchymal transition and activation of AKT signaling. *Cellular Signalling*.

[14] Dubois M., Gilles K., Hamilton J. K., Rebers P. A., Smith F. (1951). A colorimetric method for the determination of sugars. *Nature*.

[15] Bray F., Ferlay J., Soerjomataram I., Siegel R. L., Torre L. A., Jemal A. (2018). Global cancer statistics 2018: GLOBOCAN estimates of incidence and mortality worldwide for 36 cancers in 185 countries. *CA: A Cancer Journal for Clinicians*.

[16] Fang L., Zhang Y., Zang Y. (2019). HP-1 inhibits the progression of ccRCC and enhances sunitinib therapeutic effects by suppressing EMT. *Carbohydrate Polymers*.

[17] Li C., Wu X., Zhang H. (2015). A Huaier polysaccharide restrains hepatocellular carcinoma growth and metastasis by suppression angiogenesis. *International Journal of Biological Macromolecules*.

[18] Vucic D., Stennicke H. R., Pisabarro M. T., Salvesen G. S., Dixit V. M. (2000). ML-IAP, a novel inhibitor of apoptosis that is preferentially expressed in human melanomas. *Current Biology*.

[19] Zareifar S., Ghorbani S., Monabbati A. (2019). Expression of antiapoptotic proteins livin and survivin in pediatric AML patients, as prognostic markers. *Pediatric Hematology and Oncology*.

[20] Hariu H., Hirohashi Y., Torigoe T. (2005). Aberrant expression and potency as a cancer immunotherapy target of inhibitor of apoptosis protein family, Livin/ML-IAP in lung cancer. *Clinical Cancer Research*.

[21] Wang X., Xu J., Ju S., Ni H., Zhu J., Wang H. (2010). Livin gene plays a role in drug resistance of colon cancer cells. *Clinical Biochemistry*.

[22] Crnkovic-Mertens I., Hoppe-Seyler F., Butz K. (2003). Induction of apoptosis in tumor cells by siRNA-mediated silencing of the livin/ML-IAP/KIAP gene. *Oncogene*.

